# Sensing vs. seeing: body experience rather than mere body observation is linked to efficiency of descending pain modulation

**DOI:** 10.1038/s41598-026-43489-6

**Published:** 2026-04-01

**Authors:** Louisa Wolters, Benjamin Barenbrügge, Annette Löffler, Robin Bekrater-Bodmann

**Affiliations:** 1 Department of Psychiatry, Psychotherapy and Psychosomatics, Uniklinik RWTH Aachen, Aachen, Germany; 2 Scientific Center for Neuropathic Pain Aachen SCNAACHEN, Uniklinik RWTH Aachen, Roermonder Straße 110, 52072 Aachen, Germany

**Keywords:** Body perception, Deafference, Disownership, Mirror illusion, Pain inhibition, Visually induced analgesia, Neuroscience, Psychology, Psychology

## Abstract

Looking at one’s own body has been shown to exert analgesic effects on experimental pain stimulation. This study investigated the potential involvement of descending pain modulation and the role of body experience in this phenomenon. Non-clinical participants completed four experimental conditions with either a glass or mirror partition placed sagittally between their hands, which were either visible or covered with wooden boxes. This allowed participants to view either their left hand or the reflection of their right hand (each visible or covered), while they underwent a conditioned pain modulation (CPM) protocol with the test stimulus applied to their left hand. Disembodiment experiences regarding the left hand were additionally recorded using a questionnaire. Looking at the mirrored right hand significantly induced disembodiment experiences of the hidden left hand, even in the absence of explicit multisensory conflict. Across all conditions, significant CPM responses were observed. Although CPM responses were not modulated by mere vision of the left hand, their magnitude was significantly negatively correlated with disembodiment ratings. These results suggest that states of body experience modulate nociceptive processing through descending pathways. The findings are discussed within a body salience framework.

**Supplementary Information:**

The online version contains supplementary material available at 10.1038/s41598-026-43489-6.

## Introduction

Empirical findings from the past two decades provide increasing evidence for a close link between body perception and pain perception. One of the most intriguing insights is that looking at one’s own body attenuates pain^1–3^. This phenomenon, known as visually induced analgesia (VIA), was first described by Longo et al.^1^ who applied painful suprathreshold infrared laser stimuli to participants’ right hands. Looking at one’s own right hand directly, as well as looking at the reflection of one’s left hand in a sagittally placed mirror, was associated with an analgesic effect. This effect was not observed when participants looked at a non-body object or, notably, when looking at someone else’s hand, suggesting that mere vision of one’s own body reduces pain. The authors^1^ proposed that the sense of ownership for the seen hand, referring to the extent to which a limb is perceived as one’s own^4^, could be a critical determinant in this phenomenon. Beside the effects of mere visual input of the body, these and other findings have heightened interest in the importance of ownership or embodiment experiences (concepts that are often used interchangeably in the literature^4^ for pain perception. In the present study, we employ the more general term embodiment, of which ownership constitutes one dimension^5^.

Embodiment is a dynamic rather than fixed phenomenon, emerging from the continuous integration and comparison of incoming multisensory inputs with pre-existing, neurally encoded body representations^6^. A fundamental prerequisite for the emergence of embodiment, both in experimental settings and in everyday contexts, is the spatial and temporal congruence of body-related multimodal sensory inputs^7,8^. Interestingly, sensory conflicts can be used to trick these processes^6,9^, thereby inducing illusory embodiment of artificial^5^ or mirrored limbs^10^. While studies have yielded mixed results regarding the pain modulating potential of these illusions^11^, some have reported an analgesic effect^12–14^, in which embodiment experiences may play a key role^15,16 ^(for a review, see^11)^. Conversely, even small temporal or spatial incongruence in multisensory input can elicit the contrary phenomenon of disembodiment^17,18^, i.e., the feeling that one’s own body part no longer belongs to oneself, which has preliminarily been linked to enhanced, rather than reduced, pain perception^19^. In combination, these results suggest that both visual input of one’s body and subjective body experience may represent factors modulating pain perception.

But which neurophysiological mechanisms could underlie this modulation? Based on neuroimaging results, Longo et al.^2^ proposed a primarily supraspinal mechanism underlying the analgesic effect, where visual information of the body increases functional coupling between a visual body network and the broader pain network, thereby enhancing intracortical inhibition of nociceptive processes. Intracortical inhibition, however, represents only one of the mechanisms underlying endogenous pain modulation. In recent years, considerable attention has been directed toward descending pathways that contribute, for instance, to the pain modulating effects of attention or expectation^20^. These pathways involve inhibitory mechanisms mediated through the brainstem and spinal cord^21,22^, and can induce analgesic effects comparable to those observed with pharmacological analgesics^23^. Given that descending pain control is known to be modulated by cognitive processes^21,22^, it can be hypothesized that these pathways may also be affected by body perception, thereby contributing to VIA; however, to our knowledge, this hypothesis has not yet been empirically tested.

A well-established method used to probe descending pain modulation is the conditioned pain modulation (CPM) paradigm, extensively investigated as the „pain inhibits pain” phenomenon: The perceived intensity of a noxious test stimulus (TS) is modulated by a painful conditioning stimulus (CS) applied to a different body part^24^. In healthy participants, TS intensity after CS application is usually reduced compared to TS intensity before the CS^25^. Comparing TS intensity before and after the CS thus offers an indirect measure to quantify the net effect of ascending and descending pain modulatory effects^26^. At the neural level, CPM is thought to be mediated by descending inhibitory pathways, classically described as a spino-bulbo-spinal loop: noxious input activates brainstem nuclei, which in turn suppress nociceptive transmission in the dorsal horn^27^. This makes the CPM response a biomarker for descending pain modulation^28,29^. While triggered by a bottom-up stimulus (i.e., the CS), the CPM response is modulated by higher level cortical functions^30^. This premise makes it a suitable tool to test for potential effects of modulating factors on descending pain modulation and to possibly deliver an extension to the intracortical model for VIA as proposed by previous authors^2^.

Taken together, body perception (i.e., visual input of one’s body and conscious body experience) has been proposed to act as a contextual factor modulating pain perception. Whether descending pain modulation is involved, however, remains unknown. For this purpose, we combined a well-established paradigm for VIA^1^ with the CPM paradigm. The present study thus tests the hypothesis that looking at one’s body affects descending pain modulation, reflected in the efficiency of the CPM response. Furthermore, we expect a negative association between conscious body experience (i.e., disembodiment) and descending pain modulation.

## Methods

### Recruitment strategy and exclusion criteria

Participants were recruited within the academic environment and consisted primarily of students from RWTH Aachen University. As older adults have demonstrated a significantly smaller CPM response compared to younger and middle-aged adults^31^, only participants aged between 18 and 50 years were included. Individuals experiencing chronic pain, acute mental disorders, or somatic disorders with somatosensory impairments were excluded based on self-reports. Furthermore, the use of centrally active or psychotropic medication and substances was considered an exclusion criterion. Participants were instructed to abstain from the use of pain medication and alcohol for 24 h as well as of cannabis for 48 h prior to their participation. All participants had sufficient German language proficiency. The study was approved by the ethics board of the Medical Faculty of RWTH Aachen University (reference number: EK 24–210), complies with the Declaration of Helsinki in its current form, and was registered in the German Clinical Trials Register (DRKS00035677). All participants provided written informed consent prior to their participation and received monetary compensation (15€ per hour).

### Experimental design and general procedure

**Fig. 1:**
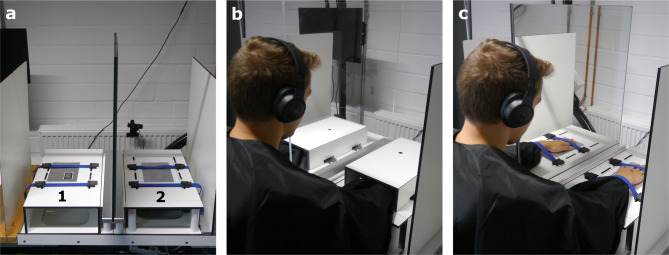
Experimental setup and conditions: Participants were seated at a table with their hands resting on two modular elements (**a**) featuring a thermal probe (1) to assess heat pain thresholds as the test stimulus (left hand) and a thermal plate (2) to induce tonic cold pain as the conditioning stimulus (right hand). The applied 2 × 2 design involved four conditions during which participants’ hands were either covered (**b**) or visible (**c**). Their hands were separated by either a glass (**b**) or a mirror partition (**c**). They were instructed to keep their head on the right side of the partition and continuously look towards their left hand while the conditioned modulation paradigm was applied. Note that the pictures b and c were staged with one of the co-authors (B.B.), who consented to their publication.

Participants were seated with both hands placed on a table equipped with custom-made modular elements (see Fig. 1a). A two-by-two factorial design with the factors *partition* (glass vs. mirror) and *hand visibility* (hands visible vs. hands covered) was employed (see Fig. 1b and c). In both *hands visible* conditions, participants’ hands were uncovered, whereas in the *hands covered* conditions, both hands were covered using custom-made wooden boxes. In each condition, participants were instructed to continuously look towards the location of their left hand. The partition was placed sagittally in front of the participants at equal distance to both hands. In the *glass* conditions, this setup allowed direct view of the left (covered or uncovered) hand through the glass partition. In the *mirror* conditions, participants looked at the reflection of their right (covered or uncovered) hand in spatial alignment with the actual position of their left hand.

The design was conceptually inspired by the VIA investigation of Longo et al.^1^, who used two visual perspectives (i.e., direct hand view vs. viewing a reflection, each compared to a neutral object) in separate experiments. In the present study, these visual perspectives were integrated into a single experimental framework, with the factor *partition* allowing us to examine potentially differential effects of visual perspective (direct view vs. reflection) on body and pain perception. The factor *hand visibility* manipulated the availability of visual body information (hands visible vs. covered), corresponding to the classical VIA paradigm. The full-factorial design allowed for the evaluation of both main effects as well as potential interactions and resulted in four experimental conditions: glass – hands visible (GV); glass – hands covered (GC); mirror – hands visible (MV); mirror – hands covered (MC). Condition order was randomized for each participant.

Prior to the four experimental runs, a test run with closed eyes was conducted to familiarize the participants with the experimental setup and procedure (the test run data was not included in analysis). This resulted in a total of five consecutive runs, each lasting approximately 10 min. The procedure was identical for all runs: It started with a two-minute induction period exposing participants to the experimental setup, which was followed by the CPM protocol (see below). After each run, a short questionnaire regarding the participants’ perception during the preceding run was administered (see below). To minimize carry-over effects of the CS, a minimum break of two minutes was implemented between runs. As each run began with the two-minute induction period, this resulted in an interval of at least 4 min between the last TS of one run and the first TS of the subsequent run.

### Experimental setup

The experimental setup is depicted in Fig. 1a. Participants placed their hands on two custom-made modular elements: a thermal plate (for application of the CS) was integrated into the right modular element, and a thermal probe (for application of the TS) was integrated into the left modular element (for technical details of the equipment, including manufacturer information, see below). To prevent intermodal sensory conflicts in the *mirror* conditions, an aluminium plate (serving as a sham thermal plate with the same dimensions; see Fig. 1a) featuring a cutout for the thermal probe was attached to the left modular element. The distance between both middle fingers was 40–42 cm, depending on the participants’ hand size. Participants were asked to remove any jewellery or watches and placed the palms of their hands on the aluminium plates with their left thenar eminence covering the thermal probe. The experimenter carefully checked that both hands were placed correctly. To ensure a stable hand position during the experiment, the hands were fixed with elastic straps (see Fig. 1c). Participants wore a black lab coat, which was tucked under the elastic straps to cover their arms.

A mirror or glass partition (experimental runs) or no additional element (test run) was placed between the left and right module (see Fig. 1). Equal distance of both hands to the mirror or glass partition was ensured using a measuring tape before each run. For the *hands covered* conditions, visual hand input was blocked by the wooden boxes (see Fig. 1b). Black dot-shaped stickers were attached to the backs of both hands as well as to a corresponding area of the two wooden boxes to indicate the direction of gaze during the experiment, and participants were instructed to continuously look in the direction of their left hand gazing the dot in view. To reduce visual input from the environment, an additional wooden partition board was placed on each outer side of the modular elements (see Fig. 1), ensuring the same neutral background in the *glass* as well as the *mirror* conditions. A foot pedal was placed directly in front of the participants’ right foot, allowing to give feedback to the thermal stimuli. To avoid accidental pressing, participants were instructed to take off their right shoe, and operating the foot pedal was practised before the test run. Participants wore wireless noise cancellation headphones to reduce distraction. A camera was installed facing the participants at a distance of approximately 55 cm from their hands. The experimenter was seated behind a privacy screen, continuously monitoring the participants’ hand placement, gaze direction, and the thermal plate’s temperature via the live camera feed.

### Hardware and software

Heat stimuli were delivered using the Thermal Cutaneous Stimulator II (TCS II, QST.Lab, Strasbourg, France), equipped with the T11 probe. The probe dimensions were 6.6 × 4.0 × 4.4 cm, and the total stimulation surface was 9 cm^2^. A thermal plate (QST.Lab, Strasbourg, France) with an aluminium stimulation area measuring 20 × 13 cm was used to induce tonic cold pain. JBL Tune 770 NC headphones (HARMAN International Industries, Northridge, CA, USA) were used. All stimuli and response recordings were programmed and controlled using PsychoPy software (v2024 2.4).

### CPM protocol and dependent variables

To minimize the influence of distraction, a sequential CPM protocol was used, as recommended for CPM research^32^. For the TS, heat pain thresholds (HPT) were assessed for the participants’ left hand before and after the CS (pre- and post-HPT, respectively), and the CPM response was calculated as the difference between post- and pre-HPT (i.e., ΔHPT). For the CS, tonic cold pain was induced to the participants’ right palm. The protocol was based on the procedure reported by Lithfous et al.^33^, and its implementation in the present study is summarized below.

#### Test stimulus (HPT)

For assessment of HPT, the method of limits was used (e.g., ^34)^. Five consecutive measurements were conducted with randomized interstimulus intervals between 2 and 4 s to reduce predictability. Starting at the baseline of 32 °C, the temperature increased at a rate of 1.5 °C/s. Participants were instructed to press the foot pedal at the very first sign of pain, regardless of its intensity. The heating process was immediately terminated upon pedal press, causing the temperature to return to baseline at a speed of 100 °C/s. HPT were calculated as the average of the last three measurements. During one HPT trial (i.e., less than 0.1% of all measurements), one participant reported having pressed the foot pedal too early; the corresponding measurement was excluded manually, and the HPT was calculated based on the remaining two measurements. For safety reasons, the maximum temperature was set to 54 °C; however, this temperature was never reached.

#### Conditioning stimulus

Immediately after the pre-HPT assessment, the CS was applied. The temperature of the thermal plate was lowered to 10 °C (i.e., the target temperature), accepting a tolerance of ± 0.5 °C based on the absolute temperature resolution indicated by the manufacturer. Once the plate reached the target temperature, it was maintained for two minutes, before returning to baseline. As any hand movement would have disrupted the mirror illusion, participants were required to keep their right hand on the thermal plate for the whole duration of each run, even when no painful stimulation of that hand took place. The thermal plate was maintained at a baseline temperature of 28 °C when no CS was applied, ensuring that no nociceptive stimulation was induced, while concurrently avoiding too long cooling durations to reach the target temperature. Crucially, HPT were assessed prior to cooling (pre-HPT) and after rewarming of the thermal plate to baseline temperature (post-HPT). The average cooling duration of the thermal plate (i.e., from baseline to target temperature) was 78.7 s (*SD* = 15.9; due to technical problems, cooling duration was recorded only in a subsample of *n* = 39 participants). Participants were instructed to press the foot pedal at the very first sign of pain after initiation of the cooling process. This allowed calculation of the average duration of painful cold stimulation (i.e., from pain onset to the initiation of rewarming of the thermal plate), which averaged 145.5 s (*SD* = 15.7, *n* = 43). Average rewarming duration from 10 °C to 28 °C was 29.0 s (*SD* = 5.4, *n* = 50).

#### Post-run questionnaire

After each run, participants rated the pain intensity of the CS on a visual analogue scale (VAS) with the anchors “no pain” and “worst imaginable pain”. Answers of the VAS were converted in values ranging from 0 to 100. Since it has been shown that painfulness of the CS predicts the CPM response^35,36^, this measure was used to evaluate the validity of the CS. Based on previous categorization of VAS pain ratings^37,38^, we defined pain induction as successful only if a rating of at least 5/100 was achieved. Ratings below this cut-off occurred in two participants, accounting for six failed CS trials out of a total of 200 trials (i.e., 3%). These participants were excluded from confirmatory analyses.

Subsequently, the participants rated six items (see Table 1) regarding the perception of their real left hand on a 7-point Likert scale ranging from − 3 = “strong disagreement” to + 3 = “strong agreement”. To allow more intuitive interpretation, responses were recoded to a positive scale from 0 to 6. The items were adapted from a questionnaire implemented by Lesur et al.^18^ to evaluate experimentally induced disembodiment and targeted two dimensions: *disownership* (describing the sensation that a body part is not one’s own) and *deafference* (describing a sensation of numbness, reduced vividness, or disappearance of a body part)^18^. Three items per dimension were presented, and the item order was randomized. For each dimension, a mean score was calculated per condition and participant.

**Table 1:** Post-run questionnaire items (adapted from Lesur et al.^18^) used to assess disembodiment on a 7-point Likert scale ranging from − 3 (strong disagreement) to + 3 (strong agreement).

	Item	Dimension
Q1	My left hand felt foreign.	Disownership
Q2	It felt as if my left hand did not belong to me anymore.	
Q3	It felt as if my left hand was not part of my body anymore.	
Q4	It felt as if my left hand had disappeared.	Deafference
Q5	My left hand felt less vivid than normal.	
Q6	My left hand felt numb.	

### Sample size calculation

The required sample size for the 2 × 2 repeated measures analysis of variance (rm-ANOVA) was estimated using G*Power (v3.1.9.7) with an alpha level of 0.05 and a power of 0.95. Since, to our knowledge, there is currently no empirical data available on the effect of body perception on the CPM response, the calculation was based on previous studies on VIA, which reported effect sizes (*f*) between 0.20 and 0.40^1,2,12,13,15,16,39,40^. Considering the novelty of our approach, a conservative effect size estimation of *f* = 0.20 was used. Assuming moderate stability of repeated CPM assessments (average correlation coefficient of approximately 0.55^41^), a required sample size of *n* = 50 participants was estimated. This sample size substantially exceeds those used in comparable studies (*n* = 15–35 participants, e.g.^1,2,12,13,15,16,39,40,42^), thus allowing for reliable statistical analyses as well as for the detection of medium-sized correlations between pain perception and body experience. Due to technical issues during the measurements (insufficient ventilation of the thermal plate), five recorded datasets could not be included in the analysis; additional participants were recruited for compensation until the target sample size of *N* = 50 was reached. However, *n* = 2 participants were excluded from all confirmatory analyses because the CS in the CPM paradigm failed (see above). This reduced the effective sample size to *n* = 48, which slightly decreases the statistical power but increases the internal validity of the study by ensuring that only participants who were successfully exposed to the CS are included in the confirmatory tests.

### Statistical analysis

Statistical analysis was performed using IBM SPSS Statistics (v29.0.2.0), except for linear mixed models (LMM), which were conducted with statistical computing software R (v4.4.0)^43^ using the *lmerTest* package^44^ and the *lmer* function. Data visualization was realized in Python (v3.14.0) using Matplotlib (v3.10.7)^45^.

#### Questionnaire data

Descriptive results are reported giving the mean (*M*), standard deviation (*SD*), median (*Mdn*) and the interquartile range (*IQR*) for each of the four experimental conditions separately. The items initially assessed two dimensions, *disownership* and *deafference.* As descriptive statistics and statistical analysis showed a similar pattern for both (see Supplementary Fig. S1 and Table S1), they were combined into the single variable *disembodiment*, defined as the mean of all six questionnaire items.

Visual inspection of questionnaire data revealed a positively skewed distribution with a clustering of low ratings, and Q-Q-plots showed deviations from normality at both tails. Thus, assumption-free LMM were chosen^46^ to test for differences in disembodiment ratings. The LMM included *partition* and *hand visibility* as fixed factors and *participant* as a random factor, allowing for variable intercepts for each participant. To test for significance of fixed effects, a type III ANOVA was conducted using Satterthwaite’s method to approximate the degrees of freedom. Where appropriate, post-hoc comparisons were conducted with simple effect analysis using *emmeans*^47^. Degrees of freedom were estimated using Kenward-Roger approximation and Bonferroni correction for multiple testing was applied by adjusting the empirical p-value (*p*_Bonf_).

#### Modulation of pain perception

To allow for the interpretation of changes in HPT relative to individual baseline levels, relative ΔHPT (rΔHPT) values were calculated, applying the following formula: (post-HPT – pre-HPT)/(post-HPT + pre-HPT). We expect this procedure to reduce the influence of interindividual differences in trait perception by standardising the ΔHPT data to baseline pain sensitivity. To improve readability, rΔHPT values were multiplied by 100 prior to reporting. As graphical inspection revealed no substantial deviation from normality, the rΔHPT data (i.e., the CPM response) was analysed using parametric tests. First, the CPM response was tested across conditions and in each condition separately by comparing rΔHPT against 0 using one-sample t-tests. Where appropriate, Bonferroni correction for multiple testing was applied by adjusting the empirical *p*-value (*p*_Bonf_).

Potential experimental modulation of pre-CS pain sensitivity, as observed in previous studies^1,2,48^, was analysed using a 2 × 2 rm-ANOVA to evaluate main effects of *partition* and *hand visibility* on pre-HPT as well as their interaction. To test for differences in CPM responses, a second 2 × 2 rm-ANOVA was performed to evaluate main effects of *partition* and *hand visibility* on rΔHPT as well as their interaction. In addition to test statistics and *p*-values, we report effect sizes as Cohen’s *d* (for t-tests) and partial eta squared *(ηp²*; for the rm-ANOVA*)*, where applicable.

Previous studies have suggested that short interstimulus intervals between repetitive thermoalgesic stimuli may lead to habituation^49^. To rule out potential order effects in our findings, we conducted a detailed descriptive inspection and a supplementary LMM analysis of the HPT data including the *time point of measurement* as an additional fixed factor. The corresponding descriptive statistics as well as a detailed description of the analyses and their results are reported in the Supplementary Materials (see Supplementary Table S2).

#### Correlation of disembodiment and CPM response

To test the hypothesis that disembodiment is associated with a reduced CPM response, correlation analyses were conducted based on the result of the LMM on disembodiment. Given the significant interaction between *hand visibility* and *partition*, we refrained from an interpretation of the significant main effect and computed two indices representing the interaction: First, an interaction term was obtained using the formula (MV + GC)/2 – (GV + MC)/2. Second, following the subsequent simple effects analysis, a difference score was calculated between the two conditions driving the interaction (MV GV). Both indices were then correlated with the corresponding rΔHPT terms. As visual inspection of histograms and Q-Q-plots indicated no substantial deviations from normality for any of these terms, Pearson correlation coefficients were calculated. Given the directional hypothesis, results are reported using one-tailed *p*-values.

## Results

### Sample description

*N* = 50 subjects participated in the experiment (25 female, 25 male participants; mean age *M* = 25.70 years; *SD* = 3.39; range: 20–38 years). Two participants (i.e., 4% of the total sample, both male) rated the CS below 5/100 (indicating an invalid CS) in at least one condition and were thus excluded from further analysis. This resulted in a final sample size of *n* = 48 participants (25 female, 23 male participants; mean age *M* = 25.52 years; *SD* = 3.34; range: 20–38 years). Of this sample, *n* = 45 participants self-identified as right-handed, and three as left-handed. All participants had normal or corrected-to-normal vision.

### Experimental manipulation of body experience: disembodiment

**Fig. 2:**
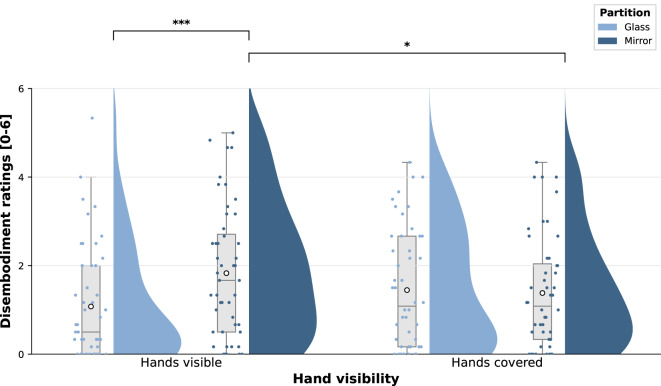
Raincloud plot of reported disembodiment ratings across conditions, with higher values indicating higher disembodiment. Boxplots: Medians and quartiles are marked by the lines of the boxes. Whiskers indicate the 1.5 inter-quartile range and ○ indicate means. Scatter plots represent individual data points and half-violins show the Kernel Density Estimates. Significance levels are indicated by *p < 0.05 and ***p < 0.001.

Disembodiment ratings are visualized in Fig. 2 (see Supplementary Fig. S1 for separate analyses of deafference and disownership ratings). At group level, participants reported strongest disembodiment in the *mirror – hands visible* condition (MV; *M* = 1.83, *SD* = 1.48, *Mdn* = 1.67, *IQR* = 2.29), and lowest in the *glass – hands visible* condition (GV; *M* = 1.08, *SD* = 1.29, *Mdn* = 0.50, *IQR* = 2.00). Ratings of both *hands covered* conditions laid in between and were comparable (*glass – hands covered*, GC: *M* = 1.45, *SD* = 1.36, *Mdn* = 1.08, *IQR* = 2.5; *mirror- hands covered*, MC: *M* = 1.38, *SD* = 1.26, *Mdn* = 1.08, *IQR* = 1.80). LMM analysis revealed a significant main effect for *partition* (*F*(1,141) = 7.777, *p* = 0.006), with *mirror* conditions being associated with higher disembodiment ratings, but no main effect for *hand visibility* (*F*(1,141) = 0.096, *p* = 0.757). Crucially, there was a significant interaction between the two factors (*F*(1,141) = 11.048, *p* = 0.001). Results of Bonferroni-corrected post-hoc tests are displayed in Table 2, showing that the interaction is mainly driven by higher disembodiment scores in the MV compared to the GV condition.

**Table 2:** Results of Bonferroni-corrected simple effect analysis of the linear mixed model for disembodiment.

	Estimate	*SE*	*df*	*t*	*p*_Bonf_
Pairwise comparisons of hand visibility by partition					
Glass: hands visible vs. covered	– 0.372	0.174	141	– 2.131	0.070
Mirror: hands visible vs. covered	0.448	0.174	141	2.569	0.022
Pairwise comparisons of partition by hand visibility					
Hands visible: glass vs. mirror	– 0.753	0.174	141	– 4.322	< 0.001
Hands covered: glass vs. mirror	0.066	0.174	141	0.378	1.000

*SE* = standard error; *df* = degrees of freedom; *p*_Bonf_ = Bonferroni-adjusted *p*-values.

### CPM response

**Fig. 3:**
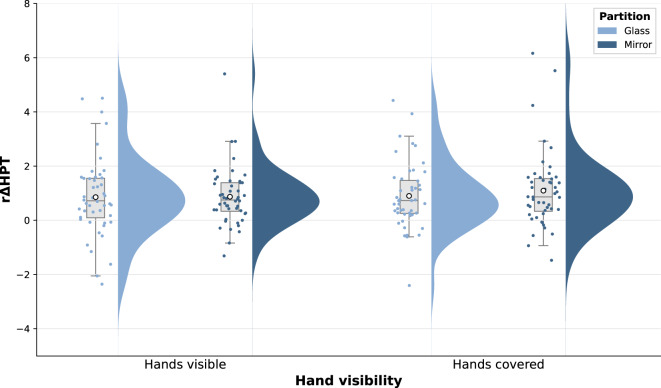
Raincloud plot of relative change in heat pain thresholds (rΔHPT) before to after the conditioning stimulus across conditions. No significant difference of the CPM response was observed between conditions. Boxplots: Medians and quartiles are marked by the lines of the boxes. Whiskers indicate the 1.5 inter-quartile range and ○ indicate means. Scatter plots represent individual data points and half-violins show the Kernel Density Estimates.

Participants (*n* = 48) reported an average CS pain intensity of *M* = 36.52/100 (*SD* = 20.69; range: 7.78–88.75). *M* and *SD* for pre-HPT, post-HPT, ΔHPT, and rΔHPT are displayed in Table 3, and rΔHPT data is visualized in Fig. 3.

Across conditions, mean rΔHPT was significantly different from 0, with a large effect size (*t*(47) = 7.834, *p* < 0.001, Cohen’s *d* = 1.131). Condition-wise one-sample t-tests revealed significant CPM responses of medium to large effect sizes each (GV: *t*(47) = 4.093, *p*_*Bonf*_ < 0.001, Cohen’s *d* = 0.591; GC: *t*(47) = 5.083, *p*_*Bonf*_ < 0.001, Cohen’s *d* = 0.734; MV: *t*(47) = 5.571, *p*_*Bonf*_ < 0.001, Cohen’s *d* = 0.804; MC: *t*(47) = 5.344, *p*_*Bonf*_ < 0.001, Cohen’s *d* = 0.771).

**Table 3:** Means (*M*) and standard deviations (*SD*) of heat pain thresholds (HPT) and changes in HPT before and after the conditioning stimulus in each condition. Note that Pearson correlation analysis between ΔHPT and rΔHPT values revealed a near-perfect positive association (*r*(46) = 0.994, *p* < 0.001, two-tailed), indicating that the relative transformation preserved the overall structure of the conditioned pain modulation data.

Condition	Pre-HPT (°C)	Post-HPT (°C)	ΔHPT (°C)	rΔHPT
	*M (SD)*	*M (SD)*	*M (SD)*	*M (SD)*
Glass – hands visible	45.90 (3.79)	46.68 (3.76)	0.78 (1.29)	0.85 (1.45)
Glass – hands covered	46.00 (3.65)	46.83 (3.65)	0.83 (1.07)	0.90 (1.23)
Mirror – hands visible	46.33 (3.67)	47.13 (3.65)	0.81 (0.96)	0.87 (1.08)
Mirror – hands covered	46.06 (3.89)	47.08 (3.88)	1.01 (1.27)	1.09 (1.42)
Mean	46.07 (3.58)	46.93 (3.64)	0.86 (0.73)	0.93 (0.82)

*M* = mean; *SD* = standard deviation; pre-HPT = heat pain threshold before conditioning stimulus; post-HPT = heat pain threshold after conditioning stimulus; ΔHPT = absolute difference in HPT (post-HPT – pre-HPT); rΔHPT = relative change in HPT.

### Experimental modulation of pre-CS pain sensitivity and CPM response

The two-way rm-ANOVA revealed no significant main effect of *partition* (*F*(1,47) = 0.243, *p* = 0.624, *ηp²* = 0.005) or *hand visibility* (*F*(1,47) = 1.839, *p* = 0.182, *ηp²* = 0.038) on pre-HPT. No significant interaction was observed between the two factors (*F*(1,47) = 0.836, *p* = 0.365, *ηp²* = 0.017).

Further, there was no significant main effect of *partition* (*F*(1,47) = 0.489, *p* = 0.488, *ηp²* = 0.010) or *hand visibility* (*F*(1,47) = 0.547, *p* = 0.463, *ηp²* = 0.011) on rΔHPT. No significant interaction was observed between the two factors (*F*(1,47) = 0.274, *p* = 0.603, *ηp²* = 0.006) regarding this measure.

### Correlation of disembodiment and CPM response

**Fig. 4:**
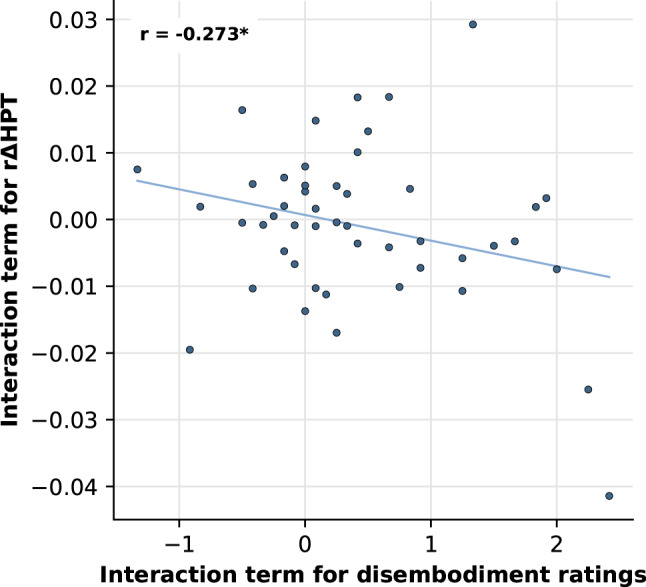
Scatter plot displaying the correlation between the interaction terms of relative change in heat pain thresholds (rΔHPT) and disembodiment ratings. Dots represent individual data points. *r* = Pearson’s correlation coefficient. The significance level is indicated by *p < 0.05 (one-tailed).

Two Pearson correlation analyses were performed to evaluate the association between disembodiment ratings and the CPM response (i.e., rΔHPT). Based on the significant interaction between *hand visibility* and *partition* observed in the LMM on disembodiment, an interaction term was computed. As post-hoc simple effect analysis revealed that this interaction was primarily driven by the significant contrast between the MV and GV conditions, a difference score between these two conditions was calculated. Both indices were correlated with the corresponding measures derived from rΔHPT. Results revealed a significant negative correlation for both the interaction term (*r*(46) = -0.273, *p* = 0.030 (one-tailed)) as well as the difference score (*r*(46) = -0.278, *p* = 0.028 (one-tailed)), the former of which is displayed in Fig. 4.

## Discussion

In this study, we probed the effect of observing one’s own painfully stimulated left hand on the CPM response in a sample of non-clinical participants. We further investigated how alterations in conscious body experience are linked to the CPM response. A significant modulation of disembodiment was observed, with the highest disembodiment ratings for the left hand being reported when participants looked at a reflection of their right hand in a mirror. Our experimental protocol induced a significant CPM response of medium to large effect size in all conditions. Statistical analysis suggests that there was no effect of mere vision of one’s own hand on the CPM response. Rather, we found a significant negative correlation between disembodiment and the CPM response. These results suggest that states of body experience are associated with the efficiency of descending pain modulation.

### Body perception and pain

Our experimental setup was based on previous studies^1,2^ investigating the effects of looking at one’s own hand while receiving above-threshold painful stimuli to the same hand. In these studies, visual input of one’s own hand (either directly viewed or viewed in a mirror) resulted in lower pain ratings and reduced cortical responses in pain-processing brain areas compared to viewing an object or another person’s hand^1,2^. The authors interpreted their results in the context of intracortical inhibition, whereby activity in a visual body network exhibits inhibitory effects on cortical processing of nociceptive information. In contrast to these findings, mere vision of one’s hand had no significant effect on the CPM response in our experiment. Although it may appear so, the two results do not necessarily contradict each other. Whereas previous studies have investigated supraspinal mechanisms of endogenous pain modulation, we examined the effects of observing one’s own body on descending pain modulation. Although the latter modulatory processes are frequently initiated at the cortical level, they exert their inhibitory influence on nociceptive transmission via brainstem and spinal cord pathways^27,50^. While mere visual input of one’s own body may modulate cortical processing of pain^2^, our results suggest that this kind of visual information does not affect the central nervous system’s fundamental nociceptive functions in terms of descending pain modulation efficiency. Our results thus highlight differential effects of body perception on distinct mechanisms of endogenous pain modulation.

Despite the lacking influence of mere visual input of one’s body, our data suggest that higher-order body perceptions are at least partly related to the efficiency of descending pain modulation. We found a significant negative relationship between disembodiment and the CPM response, which supports previous findings regarding a link between disembodiment and enhanced, rather than reduced nociceptive processing^19^. This finding may point to a novel mechanism of modulatory nociceptive processing: beside horizontal (that is, intracortical) modulation of nociceptive signals^2^, there may be additional vertical modulatory effects (that is, descending pain modulation) of conscious body experience. This is an important conclusion which must be further validated in future studies.

If prospective studies support this conclusion, body experience would be placed among a set of psychological factors or states that have previously been reported to be associated with the efficiency of descending pain modulation. It is important to note, however, that previous research regarding psychological factors modulating the CPM response has yielded mixed findings. While some studies reported associations to variables such as anxiety^51,52^, acute psychosocial stress^53^, and pain catastrophizing^54,55^, others were not able to replicate these results^56–58^. Evidence regarding the role of expectation appears more consistent: anticipated pain relief has been shown to enhance CPM responses (for a review, see^30^) and lower expected CS pain intensity has been linked to reduced CPM magnitude^59^. The present study adds to this line of research by providing initial evidence that body experience may represent an additional contextual factor modulating CPM response. However, with an amount of explained variance of less than 10%, the relationship is modest in extent so that future studies still need to elucidate the importance of our findings.

Our results are in line with preliminary evidence suggesting a hyperalgesic effect of disembodiment^19^, whereas experimentally induced embodiment experiences have been associated with analgesic effects^13,16^. In the light of our results, we propose that the *salience of the own body* may be a crucial contextual factor in this process. Previous results suggest that, under normal circumstances, the body has a low default salience level, whereas the salience level may increase when expectations about body perception are violated^60,61^. Applied to the current experiment, this would imply that multisensory-induced disembodiment of one’s own hand, due to the deviation from the ‘normal’ bodily experience, increases the salience of the affected body part. Interpreting disembodiment experiences as a state of the body characterized by enhanced salience would also be in line with predictive coding accounts, which propose that the brain continuously compares incoming sensory input with predictions based on pre-existing body representations^62^. Multisensory incongruences represent deviations from these predictions, standing out as unexpected prediction errors characterized by high levels of salience^63,64^. In the context of pain, this state of heightened alertness toward the body may manifest as a reduction in descending pain modulation, thereby increasing the likelihood of painful percepts. This could represent a useful adaptation to unusual bodily states to protect the organism from further harm in a potentially threatening situation. This new hypothesis and its implications, however, must be subjected to further empirical investigation before any conclusions regarding psychophysiological mechanisms can be drawn.

### Limitations

A first limitation concerns the applied CS (tonic cold pain induced to the palm of the hand with a thermal plate), which could be considered weaker than other commonly used stimuli (e.g., ice baths or pressure cuffs). This may have resulted in lower CS intensity ratings than those reported in previous studies (*M* = 36.52/100 vs. 44.94–72.7/100^65–67^. However, as significant CPM responses of medium to large effect sizes were observed, it appears unlikely that the null effects of mere body observation in the present study can be attributed to attenuated CPM effects. Rather, the carefully controlled CS temperature in our protocol may represent a strength compared with other CPM induction methods.

Unlike previous VIA studies might suggest^1,2,48^, we observed no experimental effect on pre-CS pain sensitivity. However, our method differed from many prior approaches by implementing HPT as the TS, whereas earlier investigations often used suprathreshold pain stimuli^1,2,48^. Evidence regarding the effects of body perception on pain detection thresholds, however, is limited^68^. The absence of a significant modulation of pre-CS HPT in the present study suggests that detection-level nociceptive processing is not substantially altered by visual body information. Importantly, the lack of this effect rules out confounding of our CPM results by experimentally induced shifts in pre-CS pain sensitivity. Future studies should systematically investigate the differential sensitivity of threshold and suprathreshold pain measures to contextual factors such as body perception.

Furthermore, methodological differences in our compared to previous studies regarding the manipulation of body experiences complicate direct comparison of the present results to the existing literature: Previous studies using similar mirror illusion setups either focused on embodiment of the reflected hand, rather than perception of the hidden, real hand^1,68^, or introduced factors directly manipulating body experience, such as visuo-proprioceptive and/or visuo-tactile mismatches^10,69^. It can be hypothesized that induction of such obvious multisensory conflicts may have elicited stronger disembodiment and, in consequence, stronger effects on descending pain modulation than the rather subtle mirror-induced alterations in our experimental setup. Nevertheless, a significant modulation of disembodiment of the real hidden hand occurred in the present study, suggesting that merely mirroring a person’s hand without further multisensory manipulation already alters the experience of one’s body. In line with body illusion studies discussing the disembodiment of the hidden real hand as a consequence of the embodiment of an artificial hand^5,70,71^, the disembodiment observed in our MV condition may have been accompanied by embodiment of the mirrored hand^13,72^. This interpretation is consistent with previous models that propose continuous updating of body representation based on incoming multisensory input^73^. In this framework, reduced confirmatory sensory input, such as the lacking visual information regarding the participants’ left hand in our experiment, may lead to disembodiment of the real body part behind the mirror^73^. This may have important implications for future studies using mirror setups for manipulation of body perception, which often assume (explicitly or implicitly) that mirrored body parts are processed like their hidden counterparts. Our findings challenge this premise, suggesting that researchers should critically evaluate the suitability of mirror setups for their research question.

A noteworthy peculiarity in the data of the present study is an increase in HPT observed over the course of the experiment (see the supplement for details). This could be due to rather short inter-condition intervals of two minutes, which were favoured to limit the overall experiment duration, as pilot testing had shown a decrease in focus and motivation with prolonged participation duration. Evidence on the duration of the CPM effect is mixed: while some studies found long-lasting effects^23,74^, others observed a normalisation of VAS ratings after 3–6 min^75^. Given this great variability and the dependence of CPM duration on specific stimulation parameters, we performed additional analyses including the time point of measurement to rule out potential carry-over effects. These analyses revealed a similar drift of pre- and post-HPT across the runs, indicating that the differences between both measures (i.e., (r)ΔHPT as a measure of the CPM response) were not substantially affected. These results are in line with those of a previous study, which reported consistent CPM responses in four consecutive runs performed within 20 min with breaks of less than two minutes between runs^76^ and support the assumption that inter-condition intervals in our experiment were sufficient. Thus, other reasons such as habituation to the repeated thermal stimulation or decrease in attention appear likely to explain the observed drift in pre- and post-HPT^49^. Nevertheless, future studies could reduce uncertainty regarding potential carry-over or habituation effects by implementing longer breaks between runs, increasing interstimulus intervals, or by introducing a control group not exposed to the CS^33^.

## Conclusion and outlook

By simultaneously assessing body perception and CPM response as an index of descending pain modulation, we integrated two research domains that, to our knowledge, have not previously been combined. Our study provides preliminary psychophysical evidence for a link between conscious body experience and descending pain modulation. Merely looking at one’s own body, however, appears to have no effect on this kind of basic nociceptive processing. Our findings support the assumption that (dis)embodiment may play a contextually modulating role in nociceptive processing and suggest an interaction with descending pain modulation. We propose that conscious body experience, and especially deviations from its default state, could act as a salient factor which may modulate the efficiency of descending pain inhibition. This interpretation, however, is preliminary and requires further validation. Future research should directly modulate embodiment experiences (e.g., by inducing visuo-proprioceptive or visuo-tactile conflicts^18,77^) to examine their causal role in CPM. If our assumptions are confirmed, body perception-related descending pain modulation may represent a complementary antinociceptive mechanism to the previously proposed intracortical inhibition model^2^. Neuroimaging approaches will be needed to identify the shared or differential neurophysiological nature underlying these two phenomena. If confirmed, our results may provide a psychophysiological basis for the clinically applied approach of correcting altered body representation^78,79^ associated with chronic pain^80,81^. If the findings can be generalized to chronic pain conditions, the effect described here could potentially be utilized to attenuate the impact of peripheral contributions to chronic pain via reinstatement of a ‘normal’ state of body experience. This clinically relevant interpretation, however, requires further investigation.

## Supplementary Information

Below is the link to the electronic supplementary material.

Supplementary Material 1

## Data Availability

In accordance with the approved ethics protocol, the data supporting the findings of the present study are not publicly available but may be obtained from the corresponding author upon reasonable request.
