# Supplementary material for: Sensing vs. seeing: body experience rather than mere body observation is linked to efficiency of descending pain modulation
Source: Sci Rep. 2026 Apr 1;16:11239. doi: 10.1038/s41598-026-43489-6 (PMC13046773; doi:10.1038/s41598-026-43489-6)
Supplement: Supplementary file 1 — Supplementary Material 1 [file 41598_2026_43489_MOESM1_ESM.pdf]

Sensing vs. seeing: Body experience rather than mere body observation is linked to efficiency of descending pain modulation

Louisa Wolters[1,2], Benjamin Barenbrügge[1,2], Annette Löffler[1,2], & Robin Bekrater-Bodmann[1,2]*

[1] Department of Psychiatry, Psychotherapy and Psychosomatics, Uniklinik RWTH Aachen, Aachen, Germany
[2] Scientific Center for Neuropathic Pain Aachen SCN[AACHEN], Uniklinik RWTH Aachen, Aachen, Germany

Supplementary material

Separate LMM analysis of disownership und deafference

The questionnaire data is visualized in Fig. S1. LMM analysis of *disownership* and *deafference* each revealed a significant main effect of *partition* (*disownership*: $F(1,141) = 9.174$, $p = 0.003$; *deafference*: $F(1,141) = 4.098$, $p = 0.045$) but not of *hand visibility* (all $F \leq 0.344$, all $p \geq 0.559$). Crucially, a significant interaction between the two experimental factors was observed in both models (*disownership*: $F(1,141) = 9.743$, $p = 0.002$; *deafference*: $F(1,141) = 9.129$, $p = 0.003$). Results of Bonferroni-corrected simple effect analyses can be found in Table S1.

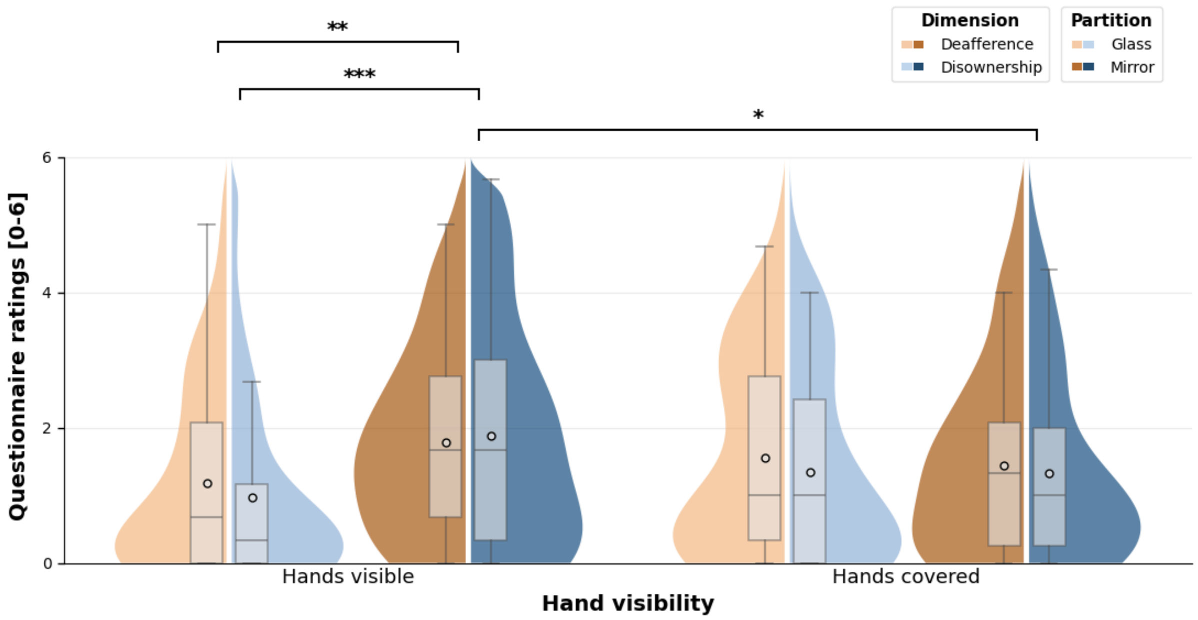

*Figure S1: Half violin plot with boxplots of reported questionnaire ratings across conditions. Each condition is represented by a two-sided composite violin: the left half depicts deafference ratings (orange) and the right half depicts disownership ratings (blue). Half-violins show the Kernel Density Estimates and densities are smoothly truncated at the upper limit of the scale to avoid sharp cut-offs. Boxplots: Medians and quartiles are marked by the lines of the boxes. Whiskers indicate the 1.5 inter-quartile range and ○ indicate means. Significance levels are indicated by * p < 0.05, ** p < 0.01, and *** p < 0.001.*

Table S1: Results of Bonferroni-corrected simple effect analysis of the linear mixed models for disownership and deafference.

	Estimate	*SE*	*df*	*t*	p_{Bonf}
Disownership					
Pairwise comparisons of hand visibility by partition					
Glass: hands visible vs. covered	-0.375	0.209	141	-1.793	0.150
Mirror: hands visible vs. covered	0.548	0.209	141	2.622	0.019
Pairwise comparisons of partition by hand visibility					
Hands visible: glass vs. mirror	-0.910	0.209	141	-4.349	< 0.001
Hands covered: glass vs. mirror	0.014	0.209	141	0.065	1.000
	Estimate	*SE*	*df*	*t*	p_{Bonf}
Deafference					
Pairwise comparisons of hand visibility by partition					
Glass: hands visible vs. covered	-0.368	0.167	141	-2.199	0.059
Mirror: hands visible vs. covered	0.347	0.167	141	2.074	0.080
Pairwise comparisons of partition by hand visibility					
Hands visible: glass vs. mirror	-0.597	0.167	141	-3.568	0.001
Hands covered: glass vs. mirror	0.118	0.167	141	0.705	0.964

Abbreviations: *SE* = standard error; *df* = degrees of freedom; p_{Bonf} = Bonferroni-adjusted *p*.

Analysis of potential order effects

Since descriptive statistics (see Table S2) suggested a linear increase in HPT over the course of the four consecutive runs, an additional LMM analysis of the HPT data was conducted. HPT were assessed twice per run (before and after the CS), resulting in a total of eight measurements per participant. The within-run factor (2 levels: pre-HPT and post-HPT) is referred to as *phase*, whereas the within-experiment factor (4 levels: run 1-4) is referred to as *measurement time point*. The LMM thus included *phase* and *measurement time point* as additional fixed factors alongside *hand visibility* and *partition,* with a random intercept for participants. To test for significant effects and interactions of the fixed factors, a type III ANOVA was conducted using Satterthwaite's method to estimate degrees of freedom.

Results revealed a significant effect of *phase* (i.e., pre- vs. post-HPT) on HPT ($F(1,321.03) = 15.2611$, $p < 0.001$), confirming our previous results regarding the CPM response. Furthermore, a significant effect of *measurement time point* on HPT ($F(1,321.07) = 8.804$, $p = 0.003$) was observed. No significant main effects of *partition* or *hand visibility* were observed (all $F < 0.876$, all $p > 0.350$) and no significant interaction effects were found between any of the fixed factors (all $F \leq 3.388$ and all $p \geq 0.067$). Crucially, no interaction of *measurement time point* and *phase* ($F(1,321.03) = 1.202$, $p = 0.274$) was observed, indicating the absence of differential order effects on either pre- or post-HPT. Taken together, these findings suggest that the CPM response remain unaffected by order effects. Furthermore, results regarding the main effects of partition and hand visibility and interactions remained consistent with the previous rm-ANOVA results regarding the CPM response.

Table S2: Means (M) and standard deviations (SD) of heat pain threshold (HPT) and changes in HPT before and after the conditioning stimulus over the course of the four consecutive runs.

Run	Pre-HPT (°C)	Post-HPT (°C)	ΔHPT (°C)	rΔHPT
	M (SD)	*M (SD)*	*M (SD)*	*M (SD)*
First	45.64 (3.36)	46.67 (3.63)	1.04 (1.19)	1.10 (1.29)
Second	46.12 (3.62)	46.96 (3.63)	0.83 (1.09)	0.90 (1.25)
Third	46.11 (3.93)	47.01 (3.86)	0.89 (1.30)	0.97 (1.50)
Fourth	46.40 (4.02)	47.08 (3.84)	0.67 (1.01)	0.74 (1.12)

Abbreviations: *M* = mean; *SD* = standard deviation; pre-HPT = heat pain threshold before conditioning stimulus; post-HPT = heat pain threshold after conditioning stimulus; ΔHPT = absolute difference in HPT (post-HPT – pre-HPT); rΔHPT = relative change in HPT.
